# Noise reduction characteristics of broadband seismometer enclosures

**DOI:** 10.1371/journal.pone.0339303

**Published:** 2025-12-23

**Authors:** Wenchao Bao, Quan An, Hao Zhai, Shubo Wang, Renkai Bao

**Affiliations:** 1 Inner Mongolia Autonomous Region Earthquake Administration, Hohhot, Inner Mongolia Autonomous Region, China; 2 Xilinhot Earthquake Monitoring Center Station, Xilinhot, Inner Mongolia Autonomous Region, China; Central South University, CHINA

## Abstract

Broadband seismometers, distinguished by their large dynamic range and wide bandwidth, have seen increasingly widespread application in earthquake early warning systems and seismological research in recent years. A quantitative investigation into the discrepancies in background noise Power Spectral Density (PSD) recorded by co-located broadband seismometers, operating with and without protective enclosures, is of substantial importance for enhancing the data quality and improving the utilization efficiency of these instruments. This paper utilizes co-located observational data from seismographic instruments (equipped with enclosures) and early warning sensors (without enclosures), installed at earthquake early warning reference stations in the Inner Mongolia region, to quantitatively investigate the noise reduction characteristics of seismometer enclosures across various frequency points, under different spatio-temporal conditions, for different components, and in diverse observational settings. The results demonstrate that subsequent to the installation of seismometer enclosures: Within the low-frequency band of 0.02–0.05 Hz, the enclosures effectively mitigate temperature fluctuations and airflow disturbances, thereby suppressing background noise. The efficacy of this suppression exhibits dependencies on both component orientation and frequency; specifically, the suppression of horizontal noise components exceeds that of the vertical component, with this noise-reducing effect becoming increasingly prominent at longer periods. The mean difference for the East-West component is 3.5 dB (median: 1 dB), while the mean difference for the vertical component is 2.2 dB. This characteristic is consistently corroborated by amplitude-squared coherence analyses performed on teleseismic event data (with the difference between the two components being approximately 0.2). Furthermore, surface-based installations benefit more significantly from such noise reduction than those situated in vaults or caves, a difference potentially attributable to the inherently greater thermal stability of subterranean environments. In the primary microseism band (0.05–0.1 Hz), the enclosures provide a discernible noise reduction effect, suggesting that the sources of primary microseisms are not solely oceanic in origin but are also modulated to some extent by the local environment proximal to the seismometer. Conversely, in the secondary microseism band (0.1–0.5 Hz) and the high-frequency band (0.5–40 Hz), the enclosures offer essentially no discernible noise reduction.

## 1. Introduction

With the formal commissioning of China’s “Earthquake Intensity Rapid Reporting and Early Warning System” (hereafter referred to as the earthquake early warning system) and the ongoing advancement of research into Earth’s fine structure, the development and utilization of broadband seismometers have become increasingly widespread [**1,2**]. The primary advantage of broadband seismometers lies in their capacity for high-precision acquisition of faint ground motion velocity in the medium to low-frequency bands [**3**], translating to a high signal-to-noise ratio recording capability for seismic signals in these bands. Noise signals recorded by broadband seismometers primarily encompass variations in temperature, humidity, and atmospheric pressure, as well as anthropogenic noise and natural environmental noise [**4–8**]. These noise sources can potentially compromise the data quality of broadband seismometers, thereby diminishing data utilization rates. Consequently, the suppression and reduction of background noise are critical for leveraging the large dynamic range and wide bandwidth characteristics of broadband seismometers.

Enhancements in the seismic signal identification and recording capabilities of seismic stations depend firstly on advanced observational equipment, secondly on scientific station siting and standardized instrument installation, and thirdly on the effective protection and maintenance of seismometers during station operation [**9**]. Seismologists conduct site surveys prior to station construction and select areas with noise levels compliant with the GB/T 19531.1–2004 standard for station deployment [**10**], thereby avoiding high-frequency noise induced by human activities, traffic, vehicles, and strong winds [**11,12**]. When the necessity arises to establish stations in high-noise environments to meet network layout requirements, borehole installations are typically chosen to mitigate both high and low-frequency background noise [**13,14**]. Subsequent to station construction, background noise is further reduced primarily through standardized seismometer installation and the addition of thermal insulation and isolation protection for the seismometers [**15,16**], thereby enhancing the quality of observational data. Previous research has indicated that temperature variations can cause changes in the geometry of the seismometer’s overall frame [**17–19**], pressure changes can induce airflow disturbances that impact the pendulum, and humid environments can readily alter the performance of the seismometer’s electronic components [**20**], ultimately diminishing the recording capability of broadband seismometers. To reduce background noise and thereby improve the ability of broadband seismometers to identify weak seismic events, seismologists generally implement thermal insulation and isolation measures for seismometers to mitigate the effects of temperature, humidity, and pressure [**21**]. What, then, are the characteristic patterns of background noise suppression when broadband seismometers are fitted with enclosures? Do the noise reduction characteristics differ across various frequency points, components, and observational methods? The primary objective of this study is to quantitatively analyze the differences in background noise Power Spectral Density (PSD) between two co-located seismometers, one with and one without an enclosure, operating during the same period, in order to ascertain the noise reduction characteristics of broadband seismometer enclosures.

**Fig 1:**
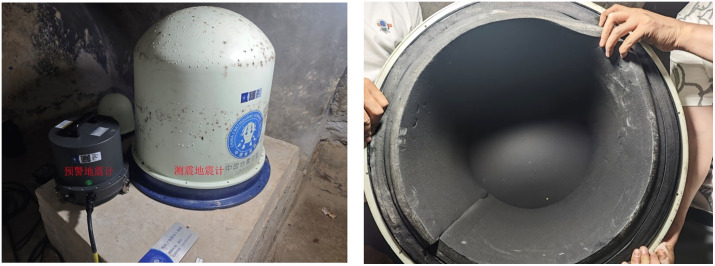
Co-located installation of early warning and seismographic seismometers on the same pier. (a) Early warning and seismographic seismometers (b) Internal structure of the seismometer enclosure.

In May 2024, seismometers (all with a bandwidth of 60 s or greater) were installed at 42 upgraded reference stations in the Inner Mongolia region, operating in real-time alongside the original seismographic seismometers. Among these, 35 stations have the early warning seismometers and seismographic seismometers strictly installed on the same pier (**Fig 1**). At these 35 stations, apart from the seismographic seismometers being equipped with enclosures while the early warning seismometers are not, all other observational conditions and seismometer performances are identical (Fig 1). As of December 2024, these 35 stations have produced continuous co-located, contemporaneous observational data from seismometers with and without enclosures, providing a rich dataset for our quantitative investigation into the noise reduction characteristics following the installation of seismometer enclosures. Based on this, the following study utilizes data from the two co-located seismometers at these 35 stations in the Inner Mongolia region, employing the Power Spectral Density (PSD) probability density function (PDF) method proposed by McNamara and Buland for the quantitative analysis of seismic background noise [**22**], to investigate the noise reduction characteristics after installing seismometer enclosures.

This study provides a reference for station construction and data quality control, offers direction for identifying interference sources in different frequency bands of observational data, and supplies a basis for installing enclosures on broadband seismometers.

## 2. Data processing and analysis

### 2.1 Data processing steps

This paper primarily employs the following steps for data processing:

(1) Data Preparation. Continuous waveform data for the year 2024 from 35 stations were downloaded from the JOPENS system of the Inner Mongolia Early Warning and Seismographic Network Center. Instrument response information for the early warning and seismographic seismometers at these 35 stations was also collected and organized.
(2) Data Preprocessing. Data segments of 1-hour length were divided into 40 recording segments, each approximately 180 s long, with a 50% overlap. These recording segments underwent detrending and mean removal. The instrument response (sensitivity) was then deconvolved to convert the recorded data values into ground velocity values.
(3) Acceleration PSD Calculation. The preprocessed recording segments were subjected to a Fast Fourier Transform (FFT) to obtain acceleration Power Spectral Density (PSD) values as a function of frequency. To facilitate comparative analysis with the global New Low Noise Model (NLNM) and New High Noise Model (NHNM), the acceleration PSD values were ultimately expressed in units of dB.
(4) Smoothing. The obtained acceleration PSD values were smoothed using 1/3 octave band smoothing, resulting in an even distribution of acceleration PSD values on a logarithmic frequency scale.
(5) PDF Value Calculation. Steps (1) through (4) were repeated to derive the hourly mean acceleration PSD distribution. Subsequently, the mean PSD distribution for the selected data segments from each station was obtained. The corresponding Probability Density Function (PDF) values for the acceleration PSDs were calculated within a range of −200 to −50 dB, using a 1 dB step. Then, a three-dimensional planar graph was generated with frequency as the abscissa, PSD as the ordinate, and color intensity representing the probability density (resulting in a PDF distribution plot). Different color blocks in this plot represent the probability of power spectra for a given frequency point falling within a specific PSD window.

This paper applies the PDF method to calculate the acceleration Power Spectral Density and corresponding Probability Density Function values for background noise data from 35 pairs of co-located seismometers, within the 0.02–40 Hz frequency band, to investigate the noise reduction characteristics of seismometer enclosures. The data processing method applied in this paper is the PDF method. In the data, normal data appears in the form of high-probability values, whereas signals such as seismic waves and surface waves that have not been manually removed, as well as instrumental spikes and calibration pulses, appear as low-probability values. These low-probability events do not affect the assessment of the high-probability background noise level.

### 2.2 Background information

**Fig 2:**
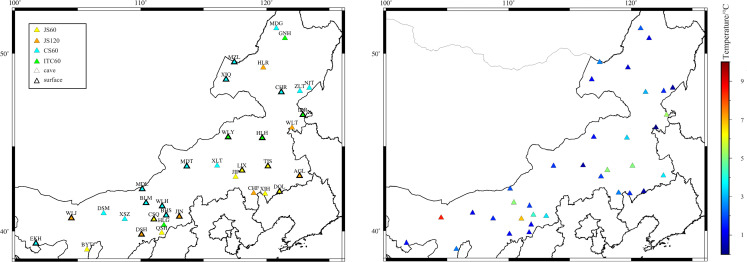
Background information. (figure was created using the GMT software, The map data is sourced from Natural Earth public domain, **http://www.naturalearthdata.com/**). **(a)** Seismometer models **(b)** Average daily temperature difference.

The selected seismometer models include the JS-60, JS-120, ITC-60, and GL-CS60 (**Fig 2a**). The sensitivity of all seismometers is 2000 ±10 v·s/m, the dynamic range is better than 140 dB, and the instrument self-noise is far below the NLNM within the studied frequency band. Therefore, its influence on the research in this paper is negligible. The observation methods are divided into cave observation and surface observation. All observation piers are built on bedrock. The average daily temperature difference of the observation environment is shown in **Fig 2b**. The cave depths are all between 50 and 100 meters, exhibiting small temperature variations. All seismometers are installed in unmanned, sealed, dedicated observation rooms.

### 2.3 PSD characteristics at different spatial locations and frequencies

**Fig 3:**
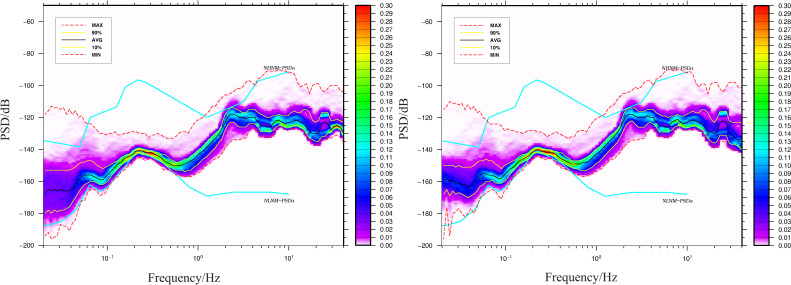
PDF and PSD of co-located seismographic and early warning seismometers. **(a)** Seismographic seismometer **(b)** Early warning seismometer.

As an example, **Fig 3** illustrates the vertical component Probability Density Function (PDF, where color intensity indicates probability) and Power Spectral Density (PSD) value distributions for the seismographic and early warning seismometers at the DSM station, covering the 0.02–40 Hz frequency band.

**Fig 4:**
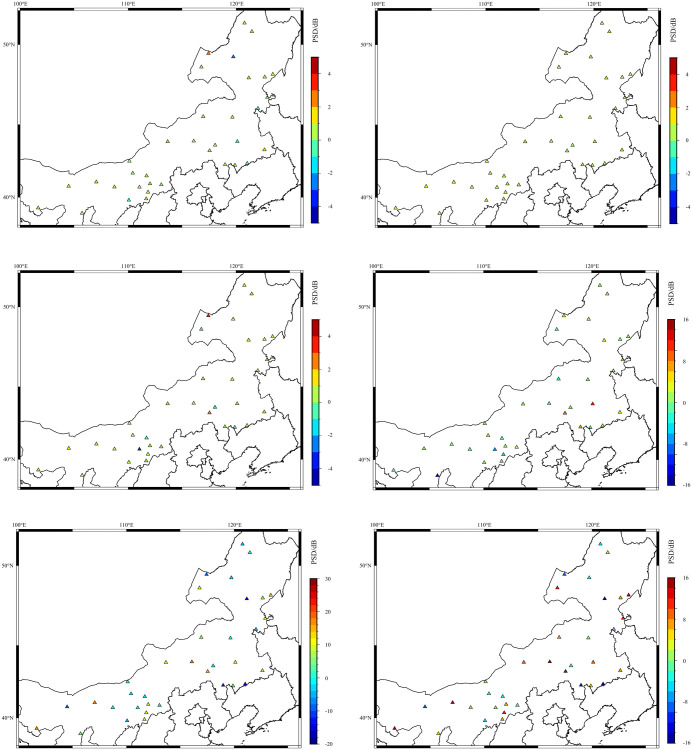
PSD differences at different spatial locations and frequency points. (figure was created using the GMT software, The map data is sourced from Natural Earth public domain, **http://www.naturalearthdata.com/**). (a) 10 Hz East-West component (b) 1 Hz East-West component (c) 0.1 Hz East-West component (d) 0.05 Hz East-West component (e) 0.02 Hz East-West component (f) 0.02 Hz Vertical component.

Recognizing that the PSD value corresponding to the maximum PDF (represented by the black line in **Fig 3**) can characterize the background noise level of the station [**10**], this value is utilized in the subsequent analysis. Fig 3 indicates that the PSD curve corresponding to the maximum PDF exhibits distinct peaks approximately within the 0.02–0.05 Hz, 0.05–0.1 Hz, 0.1–0.5 Hz, and 0.5–10 Hz frequency bands, respectively. Based on these features, the PSD values at frequency points of 0.02 Hz, 0.05 Hz, 0.1 Hz, 1 Hz, and 10 Hz were compiled for the two seismometers at all 35 stations. Furthermore, the PSD difference between the early warning seismometer and the seismographic seismometer (hereafter referred to as the PSD difference, defined as the PSD of the co-located unenclosed seismometer minus the PSD of the enclosed seismometer) was calculated for each frequency point. The spatial distribution of these PSD differences is depicted in **Fig 4**.

Fig 4(a), 4(b), 4(c), and Table 1 demonstrate that for the 10 Hz, 1 Hz, and 0.1 Hz frequency points, the percentages of PSD differences at 0 dB are 77%, 94%, and 77%, respectively. This indicates good PSD consistency at these frequency points, with the 1 Hz point showing the best consistency. Referring to Fig 4(d), 4(e), 4(f), and Table 1, at the 0.05 Hz frequency point, PSD differences registered as 0 dB in 37% of observations, while 31% of these differences were greater than 0 dB. A more notable divergence appeared at the 0.02 Hz frequency point: for the East-West component, a mere 6% of differences were at 0 dB, contrasting with 69% that exceeded 0 dB. The vertical component at this frequency, in turn, presented 14% of differences at 0 dB and 49% greater than 0 dB. This reveals poorer PSD consistency between the early warning and seismographic seismometers at the 0.05 Hz and 0.02 Hz frequency points. Furthermore, the proportion of instances where the PSD of the early warning seismometer exceeds that of the seismographic seismometer is markedly higher compared to the 10 Hz, 1 Hz, and 0.1 Hz frequency points. Additionally, at the 0.02 Hz period, the proportion of PSD differences greater than 0 dB is larger for the horizontal component than for the vertical component.

**Table 1:** Statistical characteristics of PSD differences at various frequency points.

Frequency point	10 Hz	1 Hz	0.1 Hz	0.05 Hz	0.02 Hz East-West	0.02 Hz Vertical
PSD Difference	-3dB to 2dB	0dB to 1dB	3dB to 2dB	-14dB to 11dB	-17dB to 30dB	-16dB to 16dB
Percentage of 0 dB	77%	94%	77%	37%	6%	14%
Percentage > 0 dB	8%	6%	8%	31%	69%	49%

From the spatial PSD analysis results presented above, it can be concluded that after the installation of a seismometer enclosure, the 10 Hz, 1 Hz, and 0.1 Hz frequency points, which represent high-frequency and secondary microseism bands, exhibit no noise reduction characteristics. Conversely, the 0.05 Hz and 0.02 Hz frequency points, representing primary microseism and low-frequency bands, show significant noise reduction characteristics. This conclusion aligns with the research findings of XU and YUAN (2019). The magnitude of noise reduction is greater for the horizontal component than for the vertical component, and this noise reduction feature becomes more prominent at longer periods.

### 2.4 PSD characteristics in different frequency bands

**Fig 5:**
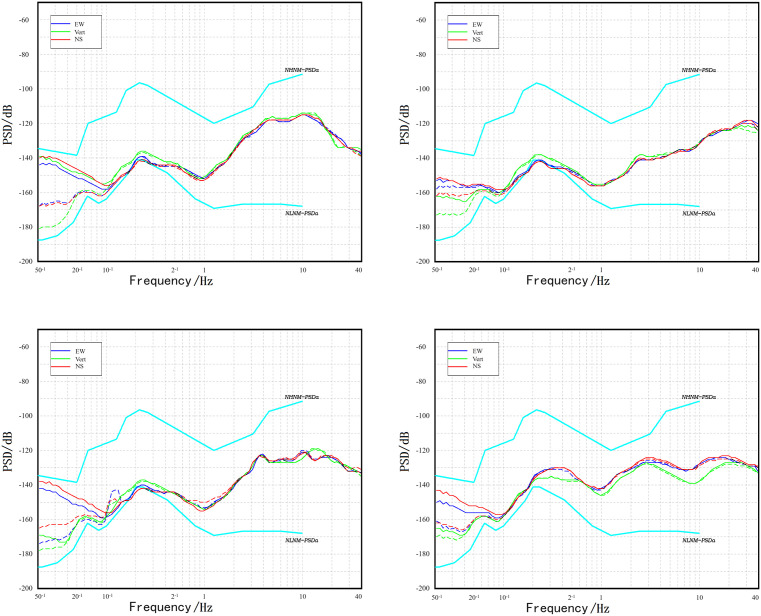
PSD distribution of seismometers in the 0.02–40 Hz band (dashed line represents the PSD of the seismographic seismometer; solid line represents the PSD of the early warning seismometer). **(a)** JIP **(b)** IDR **(c)** TIS **(d)** MDL.

Previous research has indicated that background noise sources recorded by seismometers in the high-frequency band (>1 Hz) primarily originate from anthropogenic activities [**23**]. Mid-frequency band (approximately 0.05–0.5 Hz) background noise, i.e., microseismic noise, mainly stems from the coupling action between ocean movements and the solid Earth. Within microseisms, a distinction is commonly made between primary types (spanning an approximate frequency band of 0.05–0.1 Hz) and secondary types (around 0.1–0.5 Hz) [**24,25**]. Separately, low-frequency noise, observed at periods exceeding 0.05 Hz, largely arises from localized airflow disturbances and temperature fluctuations near the seismometer [**20,21**]. Based on these research conclusions, the analysis in the following study divides the frequency spectrum into: the low-frequency band (0.02–0.05 Hz), the mid-frequency band (0.05–0.5 Hz), and the high-frequency band (0.5–40 Hz). **Fig 5** presents, as examples, the Power Spectral Density (PSD) values corresponding to the highest Probability Density Function (PDF) values as a function of frequency for four stations over the 0.02–40 Hz band.

Fig 5 demonstrates that the noise levels of the stations in the studied frequency band are all between the NLNM and NHNM. In the low-frequency (0.02–0.05 Hz) band, the shape of the noise curve for the seismometer without an enclosure is close to the NHNM, while the noise curve for the instrument with an enclosure is close to the NLNM. This suggests that the interference sources in this band may have a common origin. In the low-frequency (0.02–0.05 Hz) band and the primary microseism (0.05–0.1 Hz) band, the PSD of the early warning seismometer (unenclosed) is consistently higher than that of the co-located seismographic seismometer (enclosed). This indicates that adding an enclosure to the seismometer can suppress background noise in the low-frequency and primary microseism bands, and the magnitude of suppression increases with the period. In the secondary microseism (0.1–0.5 Hz) band and the high-frequency (0.5–40 Hz) band, the PSDs of the early warning seismometer and the seismographic seismometer exhibit very high consistency, suggesting that adding an enclosure to the seismometer does not suppress secondary microseismic noise or high-frequency noise. The conclusions drawn from the analysis of different frequency bands in this section are consistent with the conclusions from the spatial analysis in Section 2.3.

### 2.5 Time-domain characteristics of PSD at different frequency points

**Fig 6:**
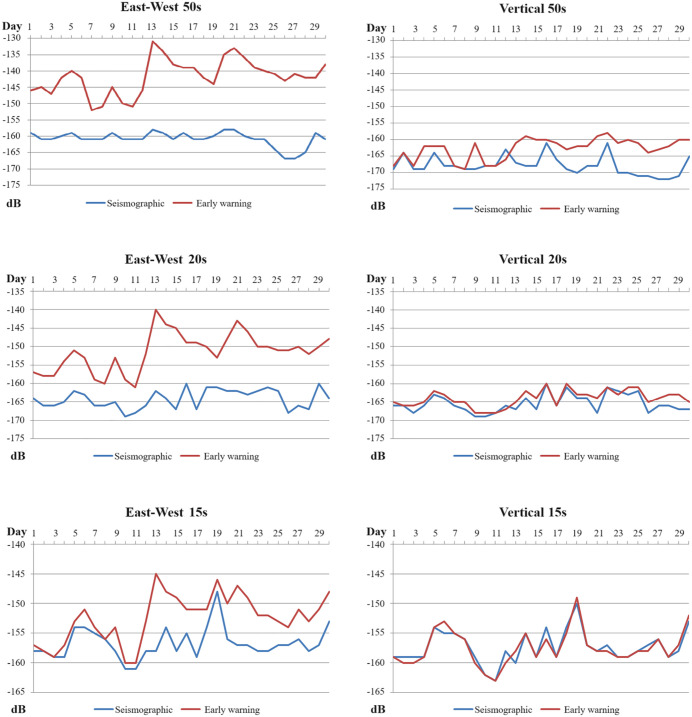
Distribution of PSD at different frequency points over time (in the figure, the vertical axis represents PSD, and the horizontal axis represents time).

To more clearly observe the time-domain variation characteristics of background noise PSD values in the low-frequency and primary microseism bands after the installation of a seismometer enclosure, the AGL station is taken as an example. Daily Power Spectral Density (PSD) values for the East-West and vertical components at the 0.02 Hz, 0.05 Hz, and 0.067 Hz frequency points were calculated for both seismometers at this station. A plot showing the distribution of these PSD values over time is presented in **Fig 6**. As can be seen, after the installation of the seismometer enclosure: at the 0.02 Hz and 0.05 Hz frequency points, representative of the low-frequency band, and at the 0.067 Hz frequency point, representative of primary microseisms, the PSD of the early warning (unenclosed) seismometer is markedly higher than that of the seismographic (enclosed) seismometer. The noise suppression characteristic due to the enclosure installation is significant. The magnitude of this suppression varies with component orientation and frequency; specifically, the magnitude of noise suppression on the horizontal component is greater than that on the vertical component, and this noise suppression feature becomes more prominent at longer periods.

### 2.6 Time-domain characteristics of PSD for different observation methods

**Fig 7:**
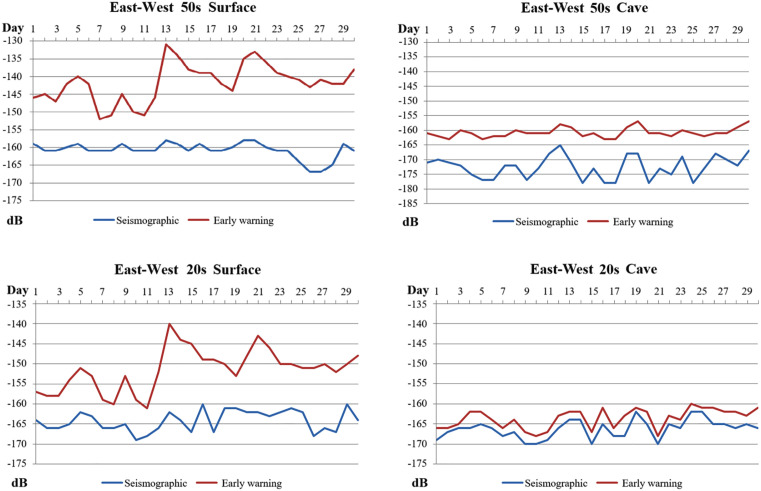
Distribution of low-frequency PSD over time for seismometers with different observation methods (in the figure, the vertical axis represents PSD, and the horizontal axis represents time). **(a)** AGL surface **(b)** DSM cave.

Taking the AGL (surface observation) station and the DSM (cave observation) station as examples, daily Power Spectral Density (PSD) values for the East-West component at the 0.02 Hz and 0.05 Hz frequency points were calculated for the early warning and seismographic seismometers at these two stations. A plot showing the distribution of these PSD values over time is presented in **Fig 7**. As can be observed, after the installation of seismometer enclosures: on a continuous time axis, for the 0.02 Hz band, the PSD reduction at the surface station reached a maximum of 27 dB and a minimum of 9 dB, while at the cave station, the PSD reduction reached a maximum of 15 dB and a minimum of 5 dB. In the 0.05 Hz band, the surface station achieved PSD reductions of 6 dB to 22 dB. The impact at the cave station was markedly less, with its noise reduction only varying between 1 dB and 4 dB.This indicates that after the installation of seismometer enclosures, the PSD reduction at surface observation stations is significantly greater than at cave observation stations. We speculate that this may be related to the inherently better constant temperature and pressure characteristics of cave observation environments.

**Fig 8:**
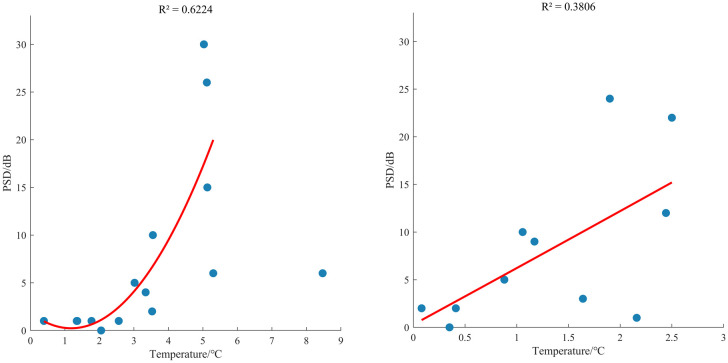
PSD difference at 0.02 Hz under different station daily mean temperature differences. **(a)** Surface **(b)** Cave.

In conjunction with Fig 2b, the correlation between the noise reduction magnitude at surface-based stations and the amplitude of environmental temperature fluctuations is more significant compared to that at cave stations (**Fig 8**). This is potentially attributable to the smaller temperature variations observed at cave stations.

### 2.7 Coherence function estimation

**Fig 9:**
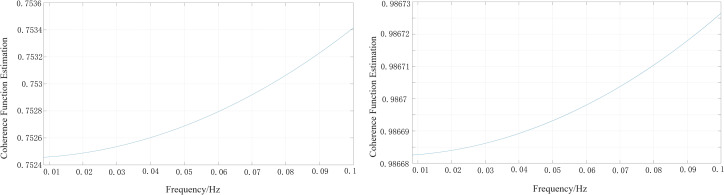
Coherence coefficients of teleseismic records from the two seismometers. **(a)** East-West **(b)** North-South.

Observational data from the Ms 7.2 Vanuatu Islands earthquake (17.75°S, 167.95°E, epicentral distance 8000 km), which occurred at 09:47 on December 17, 2024, recorded by the seismographic and early warning seismometers at the AGL station, were selected. The squared amplitude coherence coefficients were calculated for the 0.01–0.1 Hz frequency band to quantitatively evaluate the degree of matching of the teleseismic recordings in the frequency domain [**26**]. As seen in **Fig 9**, the squared amplitude coherence coefficient for the East-West component is markedly lower than that for the vertical component. Both components exhibit the characteristic of decreasing coherence coefficient with increasing period. This is consistent with the finding that after seismometer enclosure installation, the suppression of horizontal noise amplitude is greater than that for the vertical component.

## 3. Conclusions

This paper utilized data produced in 2024 from co-located seismographic and early warning seismometers in the Inner Mongolia region to investigate the noise reduction characteristics after installing seismometer enclosures, from the perspectives of different frequency points, spatio-temporal conditions, component orientations, and observation methods. The following conclusions were obtained:

1) In the low-frequency band, the installation of a seismometer enclosure can effectively suppress temperature variations and barometric pressure disturbances, thereby reducing low-frequency background noise. The magnitude of this suppression varies with component orientation and frequency; specifically, the suppression of horizontal noise is greater than that for the vertical component. At 0.02 Hz, the proportion of PSD differences greater than 0 dB for the East-West component reached 69%, which was 20% higher than that of the vertical component (the mean difference for the East-West component is 3.5 dB, median: 1 dB; the mean difference for the vertical component is 2.2 dB, median: 0 dB). This noise suppression feature becomes more prominent at longer periods. Taking the AGL station as an example, the co-located East-West component differences at 0.067 Hz, 0.05 Hz, and 0.02 Hz can reach 5 dB, 10 dB, and 20 dB, respectively. The background noise reduction at surface observation stations is greater than at cave observation stations. Taking the AGL and DSM stations as examples: for the 0.02 Hz band, the PSD reduction at the surface station reached a maximum of 27 dB and a minimum of 9 dB, while at the cave station, the PSD reduction reached a maximum of 15 dB and a minimum of 5 dB. For the 0.05 Hz band, the PSD reduction at the surface station reached a maximum of 22 dB and a minimum of 6 dB, while at the cave station, the PSD reduction reached a maximum of 4 dB and a minimum of 1 dB. This may be related to the inherently more stable thermal and barometric conditions of cave (or vault) environments. However, overall, the correlation between noise suppression (after enclosure installation) at surface-based stations and temperature is higher than that at cave stations.
2) In the mid-frequency band, after the installation of a seismometer enclosure, there is a discernible noise reduction effect in the primary microseism (0.05–0.1 Hz) band. Taking the AGL station as an example, the co-located East-West component difference at 0.067 Hz can reach 5 dB. This suggests that sources of primary microseismic noise are not solely oceanic in origin but are also influenced to some extent by the local environment near the seismometer. After enclosure installation, over 77% of stations show a PSD difference of 0 dB in the secondary microseism (0.1–0.5 Hz) band, and the number of stations with positive and negative differences is similar. This indicates no noise reduction effect in the secondary microseism band, which is consistent with the far-field noise characteristics and probability distribution of signals originating from oceanic activity.
3) In the high-frequency band, the installation of a seismometer enclosure does not provide any noise reduction, which is consistent with the characteristic that high-frequency noise sources are primarily anthropogenic.
4) In teleseismic records, the squared amplitude coherence coefficient for the East-West component is significantly lower than that for the vertical component, with both components exhibiting a trend of decreasing coherence as the period increases.

Reducing background noise can enhance the data quality of observations, which in turn improves the accuracy of earthquake early warning and seismological research. Therefore, research related to the effective protection of seismometers and data quality enhancement is highly necessary. Consequently, based on the characteristic that seismometer enclosures can suppress primary microseisms and low-frequency background noise, we recommend that seismometers at surface observation stations monitoring the frequency band below 0.05 Hz be fitted with enclosures to improve observational data quality. For cave stations (or vaults), the addition of enclosures can be considered based on specific conditions.

The research findings of this paper provide a basis for underscoring the importance of installing enclosures on broadband seismometers. Furthermore, this practice represents an important means for enhancing data quality and improving data utilization rates at existing and future fixed and mobile broadband and broader-band seismic stations.
